# One-year versus three-year outcomes of DK-Crush versus Culotte for left main bifurcation lesions: A systematic review and meta-analysis of RCTs

**DOI:** 10.21542/gcsp.2025.46

**Published:** 2025-10-31

**Authors:** Arga Setyo Adji, Intan Komalasari, Diski Saisa, Atiyatum Billah, Nadhifa, Antonius Dwi Saputra, Derren David Homenta Rampengan, Yudi Her Oktaviano

**Affiliations:** 1 Department of Cardiology, Universitas Hang Tuah, Surabaya, Indonesia; 2 Faculty of Medicine, Universitas Indonesia, Jakarta, Indonesia; 3 Faculty of Medicine, Universitas Hang Tuah, Surabaya, Indonesia; 4 Faculty of Medicine, University of Jember, Jember, Indonesia; 5 Department of Cardiology and Vascular Medicine, Airlangga University, Surabaya, Indonesia; 6 Faculty of Medicine, Universitas Sam Ratulangi, Manado, Indonesia

## Abstract

Background: The long-term comparative effectiveness of the Double Kissing (DK) Crush and Culotte stenting techniques for left main bifurcation lesions (LMBLs) remains a subject of clinical debate. This meta-analysis aimed to evaluate and compare the one-year and three-year clinical outcomes of the DK Crush and Culotte techniques in patients with LMBLs.

Methods: A systematic search was performed in PubMed, MEDLINE, Cochrane, ScienceDirect, and Google Scholar databases up to June 2025. Three randomized controlled trials (RCTs) comprising 1,188 patients with LMBLs were included. Pooled risk ratios (RRs) with 95% confidence intervals (CIs) were calculated using the Mantel–Haenszel fixed-effects model to assess one- and three-year outcomes, including target vessel revascularization (TVR), major adverse cardiac events (MACE), myocardial infarction (MI), cardiac death (CD), and coronary artery bypass grafting (CABG).

Results: Across all studies, DK Crush demonstrated significantly improved outcomes compared with the Culotte technique for several endpoints. At the one-year follow-up, DK Crush was associated with a significantly lower rate of target vessel revascularization (RR = 0.42, 95% CI: 0.24–0.75, *p* = 0.003) and major adverse cardiac events (RR = 0.45, 95% CI: 0.29–0.71, *p* = 0.0006). There were no significant differences between the two techniques in cardiac death (RR = 0.83, 95% CI: 0.32–2.12, *p* = 0.70), myocardial infarction (RR = 0.60, 95% CI: 0.26–1.39, *p* = 0.24), or coronary artery bypass grafting (RR = 2.42, 95% CI: 0.43–13.69, *p* = 0.32). At the three-year follow-up, DK Crush maintained its superiority, showing significantly reduced rates of TVR (RR = 0.37, 95% CI: 0.24–0.58, *p* < 0.0001), MACE (RR = 0.47, 95% CI: 0.34–0.65, *p* < 0.0001), and MI (RR = 0.43, 95% CI: 0.21–0.89, *p* = 0.02). However, no statistically significant differences were observed in cardiac death (RR = 0.81, 95% CI: 0.45–1.46, *p* = 0.47) or CABG (RR = 2.25, 95% CI: 0.46–11.03, *p* = 0.32).

Conclusion: The DK Crush technique demonstrated superior clinical efficacy compared with the Culotte technique, with significantly lower rates of TVR, MACE, and MI at both one and three years. Although no significant differences were observed in cardiac death or CABG, DK Crush appeared to provide more favorable long-term vessel patency and reduced risk of repeat revascularization. Further large-scale randomized trials are warranted to validate these findings and strengthen the evidence supporting DK Crush as the preferred strategy for complex left main bifurcation lesions.

## Introduction

Coronary bifurcation lesions, particularly those involving the distal unprotected left main coronary artery (ULMCA), represent some of the most technically challenging scenarios in percutaneous coronary intervention (PCI). These lesions, typically classified using the Medina system (e.g., 1,1,1; 1,0,1; or 0,1,1), require careful assessment of disease distribution across the main and side branches to determine the optimal stenting strategy. Among the available two-stent techniques, double-kissing (DK) crush and culotte stenting have become the most frequently applied and compared approaches for complex bifurcation disease^1^.

The DKCRUSH-III trial, a multicenter randomized study of 419 patients with distal left main bifurcation lesions (LMDBLs), demonstrated that compared with culotte stenting, DK crush significantly reduced 3-year MACE rates (8.2% vs. 23.7%; *p* < 0.001), with a 0% incidence of stent thrombosis versus 3.4% in the culotte group^1^.

Furthermore, patients with complex lesions according to the DEFINITION criteria had disproportionately worse outcomes with culotte (51.5% MACE) than with DK crush (15.1%; *p* < 0.001). These findings suggest that the stenting strategy plays a critical role in long-term safety and efficacy in this high-risk population^1,2^.

Long-term follow-up data from other studies, such as the DKCRUSH-V study, further confirmed the superiority of the DK crush method over provisional and other two-stent methods. According to a 3-year analysis involving 482 patients, DK crush reduced target lesion failure (TLF) to 8.3% compared with 16.9% in the provisional group (*p* = 0.005), with a stent thrombosis rate of only 0.4% versus 4.1% (*p* = 0.006)^3,4^.

A separate network meta-analysis including 7,257 patients across 26 randomized controlled trials revealed that DK crush was the top-performing technique for reducing MACE (OR 0.40 vs. culotte, 95% CI: 0.26–0.60) and target lesion revascularization^4,5^. The procedural differences between the two techniques may lead to distinct long-term outcomes. DK crush involves systematic double kissing balloon inflations and intentional side branch re-access after initial crush, potentially improving stent expansion, ostial coverage, and flow symmetry at the carina. In contrast, the culotte technique provides full coverage of both branches with a more symmetrical double-layered segment but may carry a higher risk of stent overlap, malapposition, or neocarina formation. These mechanical and geometric distinctions form the biological rationale for expecting differences in restenosis, thrombosis, and vessel patency between the two strategies.

Despite these findings, real-world data from registries such as PROGRESS-BIFURCATION suggest that culotte stenting is still used in more than 30% of bifurcation PCIs, including complex LM lesions, even though DK crush is associated with significantly lower MACE (HR 0.28; 95% CI: 0.13–0.60; *p* = 0.001) and target vessel revascularization (14.3% vs. 29.3%, *p* = 0.029)^6^. The ongoing use of culotte despite these outcomes reveals a critical gap between evidence and practice. Therefore, a comprehensive systematic review and meta-analysis comparing one- and three-year outcomes of DK crush versus culotte stenting is necessary to inform future guidelines and improve patient care in complex bifurcation PCI^5,7,8^. Although several randomized controlled trials (RCTs), such as the DKCRUSH series, have reported favorable outcomes for DK crush in specific settings, results across studies remain inconsistent. For instance, while DKCRUSH-III and DKCRUSH-V suggested lower rates of major adverse cardiac events (MACE) and stent thrombosis with DK crush, other analyses and registries have failed to confirm a clear long-term survival or revascularization advantage. Moreover, culotte stenting continues to be widely used in real-world practice, accounting for over 30% of bifurcation PCIs, reflecting persistent uncertainty regarding the true magnitude and durability of benefit between techniques.

Given these mixed data, the specific knowledge gap lies in the absence of a consolidated assessment comparing both one-year and three-year clinical outcomes of DK crush versus culotte stenting in distal left main bifurcation lesions (LMBLs). Addressing this gap is critical for defining the optimal strategy for complex LM bifurcation PCI and informing guideline recommendations. Therefore, this systematic review and meta-analysis was conducted to comprehensively compare the short- and long-term efficacy and safety of DK crush and culotte techniques, integrating evidence from randomized controlled trials.

## Methods

This systematic review was developed in accordance with the PRISMA guidelines^9^. It is registered with the number CRD420251064878 in the International Prospective Register of Systematic Reviews (PROSPERO).

### Eligibility criteria

For this systematic review and meta-analysis, we applied predefined eligibility criteria to ensure the consistency and quality of the included studies. The target population consisted of adult patients aged 18 years or older who had left main coronary artery (LMCA) bifurcation lesions classified as Medina 1,1,1 or 0,1,1, with follow-up periods of 12 months and 36 months. Only randomized controlled trials (RCTs) were considered eligible. The inclusion criteria were as follows: (1) studies involving adult patients (≥18 years) with angiographically confirmed LMCA bifurcation lesions classified as Medina 1,1,1 or 0,1,1; (2) studies that directly compared DK-Crush stenting with Culotte stenting techniques; (3) studies that reported at least one clinical outcome, including major adverse cardiac events (MACE), cardiac death (CD), myocardial infarction (MI), coronary artery bypass grafting (CABG), and target vessel revascularization (TVR); (4) studies published in English; and (5) randomized controlled trials only. The exclusion criteria were as follows: (1) studies that did not include a direct comparison between the DK-Crush technique and Culotte technique; (2) studies lacking relevant clinical outcome data; (3) nonrandomized studies such as observational studies, case series, case reports, or review articles; and (4) studies that were not published in English or did not provide full-text access.

### Search strategy and selection of studies

A comprehensive literature search was conducted to identify all relevant studies published up to June 2025 across multiple electronic databases. These included PubMed, ScienceDirect, Google Scholar, Europe PMC, Embase, MEDLINE, and the Cochrane Library. The search was designed to capture randomized controlled trials (RCTs) comparing the DK-Crush and Culotte stenting techniques in patients with left main coronary artery (LMCA) bifurcation lesions. The key search terms used included combinations of “DK-Crush”, “Culotte”, “Left Main Coronary Artery”, “Bifurcation”, “Medina 1,1,1”, and “Medina 0,1,1”, combined with outcome-related terms such as “MACE”, “cardiac death”, “MI”, “TVR”, and “CABG”. Boolean operators “AND” and “OR” were applied to optimize the search strategy and enhance sensitivity and specificity. The reference lists of the included studies and relevant systematic reviews were manually screened to identify additional eligible trials not captured through the initial database search.

### Data extraction

Designated investigators (I.K., A.S.A., A.D.S., A.B., D.S., and N.N.D.) meticulously extracted relevant data via a standardized data extraction form following the identification of eligible studies. The extracted data encompassed detailed study characteristics, including author names, year of publication, study design, study location (country), and study period. Population characteristics, such as the mean age ± standard deviation (S.D.), number and percentage of male participants, bifurcation classification (Medina 1,1,1 or 0,1,1), and the presence of cardiovascular risk factors, including diabetes mellitus (DM), hypertension (HT), smoking status, and hyperlipidemia, were also recorded. Interventional details included the type of stent used (DK-Crush or Culotte), duration of dual antiplatelet therapy (DAPT), final kissing balloon dilation (FKBD) percentage, mean vessel length (MVL), side branch length (SBL), mean vessel diameter (MVD), and side branch diameter (SBD). To ensure the accuracy and integrity of the data extraction process, an independent investigator conducted a thorough cross-check of the extracted data, verifying completeness and consistency. This validation step enhanced the reliability and robustness of the data used for subsequent meta-analysis.

### Quality assessment

For quality assessment, the GRADE (Grading of Recommendations, Assessment, Development and Evaluation) approach was applied to evaluate the certainty of evidence across five domains: risk of bias, inconsistency, indirectness, imprecision, and publication bias. Each included RCT was independently assessed by three reviewers (A.S.A., A.D.S., and A.B.). Discrepancies were discussed and resolved by consensus, with arbitration by a senior reviewer (I.O, and Y.H.O) when necessary. Data extraction was performed independently by six reviewers working in pairs, ensuring accuracy and reproducibility. Inter-reviewer disagreements regarding extracted data or quality scores were resolved through consensus discussion^10^.

### Outcome measures

The analysis encompassed a range of outcome measures, including major adverse cardiac events (MACEs), cardiac death (CD), myocardial infarction (MI), target vessel revascularization (TVR), and coronary artery bypass grafting (CABG). These endpoints were selected to provide a comprehensive evaluation of patient prognosis and interventional efficacy.

### Data synthesis and statistical analysis

Each outcome measure was analyzed by calculating the pooled risk ratio (RR) along with the corresponding 95% confidence interval (CI) via standard meta-analytic methods. The I^2^ statistic was employed to evaluate heterogeneity across the included studies. The outcomes assessed included major adverse cardiac events (MACEs), cardiac death (CD), myocardial infarction (MI), target vessel revascularization (TVR), coronary artery bypass grafting (CABG). To test the robustness of the pooled estimates, a sensitivity analysis was performed. Statistical significance was defined as a *p* value of less than 0.05. All the statistical analyses were conducted via RStudio^11^.

## Results

### Study selection, process, and quality assessment

**Figure 1.**
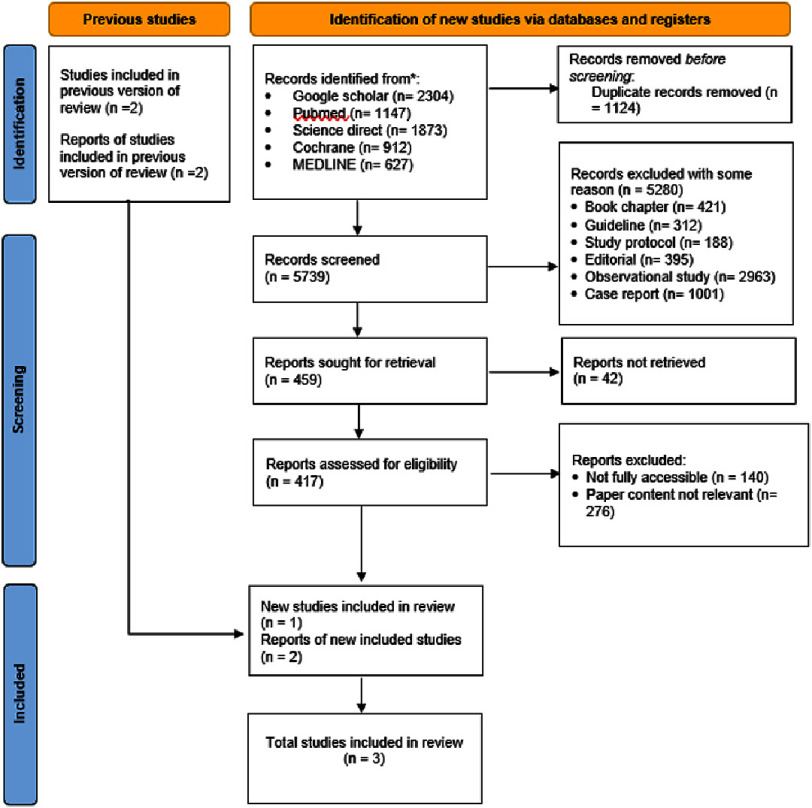
PRISMA flow diagram of the study selection process.

A comprehensive literature search was conducted across five major databases—Google Scholar, PubMed, ScienceDirect, Cochrane Library, and MEDLINE—covering studies published up to June 2025. In total, 6,863 records were initially identified, along with 6 additional records retrieved through citation searches. After removing 1,124 duplicates, 5,739 records underwent title and abstract screening. Of these, 5,280 were excluded for the following reasons: book chapters (*n* = 421), guidelines (*n* = 312), study protocols (*n* = 188), editorials (*n* = 395), observational studies (*n* = 2,963), and case reports (*n* = 1,001). A total of 459 full-text articles were sought for retrieval; 42 could not be accessed due to subscription restrictions or unavailable full texts. The remaining 417 reports were reviewed in detail. Of these, 140 were classified as non-accessible (due to paywall, missing supplementary data, or incomplete publication), and 276 were excluded for irrelevant content (did not directly compare DK-Crush and Culotte or lacked outcome data). A supplementary table (Table S1) lists all 140 non-accessible studies with their exclusion reasons for transparency. Ultimately, three randomized controlled trials (RCTs) met all inclusion criteria and were included in the final quantitative synthesis. The entire study selection process is illustrated in the PRISMA 2020 flow diagram (Figure 1).

### Study characteristics

Table 1 provides data from two studies involving 418 patients with coronary bifurcation lesions. Three of these studies were randomized controlled trials. The study populations were from China and the United States, with 208 patients from China and 119 from the United States. The mean age of the participants in the studies was 64.3 ± 10.3 years. The patient populations are diverse, with diabetes mellitus (DM) affecting 31.9% and 33.8% of the patients, hypertension (HT) affecting 70.5% and 73.6%, and smoking affecting 27.6% and 19.5% in the two studies, respectively. The majority of patients in both studies received a 1st-2nd DES (drug-eluting stent). The material parameters included a mean vessel length (MVL) of 33.48 ± 14.01 mm and 35.74 ± 15.99 mm, a stent balloon length (SBL) of 25.90 ±13.83 mm and 26.72 ± 11.86 mm, a mean vessel diameter (MVD) of 3.40 ± 0.34 mm and 3.34 ± 0.40 mm, and a stent balloon diameter (SBD) of 3.04 ± 0.41 mm and 3.03 ± 0.41 mm. The studies had a follow-up period of 12 months, and the duration of dual antiplatelet therapy (DAPT) was 12 months for both studies. The data presented in the table show the clinical characteristics and stent parameters used in the analysis of the left main bifurcation lesions in the patient cohort^1,6,12^.

**Table 1:** Patient characteristics.

		**Chen 2015**	**Chen 2013**	**Mutlu 2025**
**Study design**		RCT	RCT	RCT
**Country**		China	China	US
**Study period**		3yrs	1yr	3yrs
**Population**	***DK Crush***	208	210	119
	***Culotte***	207	209	58
**Type of stent**		1st-2nd DES	1st-2nd DES	N/A
**Mean age ± S.D**		64.3 ± 10.3/63.3 ± 9.2	64.3 ± 10.3/63.3 ± 9.2	67.50 ± 10.75/66.39 ± 10.09
**Procedural success**		DK: 98.6% vs Culotte: 97.8% (ns)	DK: 96.7% vs Culotte: 96.2% (*p* = 0.80)	DK: 96.5% vs Culotte: 95.1% (*p* = 0.698)
**Procedural time (mins)**		DK: Not reported (slightly longer than Culotte)	DK: 56.9 ± 33.1 vs Culotte: 54.9 ± 32.1 (*p* = 0.53)	DK: 113 [79–174] vs Culotte: 70 [51–102] (*p* < 0.001)
**Fluoroscopy time (mins)**		DK: Not reported	DK: 26.6 ± 14.4 vs Culotte: 27.7 ± 17.5 (*p* = 0.49)	70 [51–102] (*p* < 0.001)
** **				DK: 30 [22–44] vs Culotte: 24 [16–36] (*p* = 0.004)
**Man, n (%)**		162 (77.1)/167 (79.9)	162 (77.1)/167 (79.9)	112 (75.7)/47 (70.1)
**Medina (%)**	***1.1.1***	207 (98.7)/198 (94.8)	207 (98.7)/198 (94.8)	121 (75.2)/45 (63.4)
	***0.1.1***	3 (1.3)/11 (5.2)	3 (1.3)/11 (5.2)	21 (13.0)/16 (22.5)
**DM (n)**		31.9 (67)/30.1 (63)	31.9 (67)/30.1 (63)	33.8 (50)/37.3 (25)
**HT (n)**		70.5 (148)/61.2 (128)	70.5 (148)/61.2 (128)	73.6 (109)/79.1 (53)
**Smoking (n)**		27.6 (58)/25.8 (54)	27.6 (58)/25.8 (54)	19.5 (29)/19.4 (13)
**Hyperlipidemia (n)**		41.4 (87)/42.1 (88)	41.4 (87)/42.1 (88)	81.8 (121)/85.1 (57)
**DAPT periods**		12 months	12 months	N/A
**FKBD %**		209 (99.5)/208 (99.5)	209 (99.5)/208 (99.5)	N/A
**Stent (mm)**	***MVL***	33.48 ± 14.01/35.74 ± 15.99	33.48 ± 14.01/35.74 ± 15.99	10.00 ± 7.41/9.50 ± 3.70
	***SBL***	25.90 ± 13.83/26.72 ± 11.86	25.90 ± 13.83/26.72 ± 11.86	10.00 ± 6.48/10.00 ± 3.70
	***MVD***	3.40 ± 0.34/3.34 ± 0.40	3.40 ± 0.34/3.34 ± 0.40	3.00 ± 0.37/3.00 ± 0.37
	***SBD***	3.04 ± 0.41/3.03 ± 0.41	3.04 ± 0.41/3.03 ± 0.41	2.75 ± 0.37/2.50 ± 0.19

**Notes.**

Abbreviations CABG Coronary Artery Bypass Grafting CD Cardiac Death DS Diameter Stenosis DK Crush Double Kissing Crush LAD Left Anterior Descending LCX Left Circumflex Artery MLD Minimal Lumen Diameter MACE Major Adverse Cardiac Events MI Myocardial Infarction TVR Target Vessel Revascularization

### Risk of bias

The overall quality assessment of the included studies was evaluated using both the the GRADE framework, which rated the certainty of evidence for each outcome as low to moderate (Table 2). Two of the three randomized controlled trials demonstrated generally low risk of bias, while one study showed some concern related to allocation concealment and blinding of outcome assessment. Outcomes such as myocardial infarction (MI) and cardiac death (CD) were rated as having moderate certainty, reflecting consistent findings and precise estimates, whereas CABG, MACE, and TVR were rated as low due to downgrades for potential inconsistency and imprecision. The downgrading was primarily driven by small sample sizes and event counts, which may have introduced uncertainty in effect estimates.

**Table 2:** Summary of results and certainty assessment. Reproduced from Komalasari I, Adji AS, Billah A et al. Long-Term Outcomes of Crush versus Culotte Stenting in Coronary Bifurcation Lesions: A Systematic Review and Network Meta-Analysis [version 1; peer review: awaiting peer review]. F1000Research 2025, 14:823 (https://doi.org/10.12688/f1000research.168528.1).

**Endpoint**	**DK-Crush vs. Culotte in LMBL**					
	**DK-Crush (event/total)**	**Culotte (event/total)**	**RR (95% CI)**		**p value**	**Certainty (GRADE)**
			**One-year**	**Three-year**		
CD	34/656	24/532	0.83 (0.32, 2.12)	0.81 (0.49, 1.34)	0.42	⨁⨁⨁○ Moderate^a^
CABG	12/656	3/532	2.42 (0.43, 13.69)	2.25 (0.46, 11.03)	0.32	⨁⨁○○ Low^a^,^b^
MI	20/656	34/532	0.60 (0.26, 1.39)	0.43 (0.21, 0.89)	0.01	⨁⨁⨁○ Moderate^b^,^c^
MACE	80/654	122/530	0.45 (0.29, 0.71)	0.47 (0.34, 0.65)	<0.00001	⨁⨁○○ Low^a^,^b^
TVR	47/654	88/532	0.42 (0.43, 13.69)	0.37 (0.24, 0.58)	<0.00001	⨁⨁○○ Low^a^,^b^

**Notes.**

a Downgraded by one level for serious risk of bias.

b Downgraded by one level for serious inconsistency.

c Downgraded by one level for serious imprecision.

**Figure 2.**
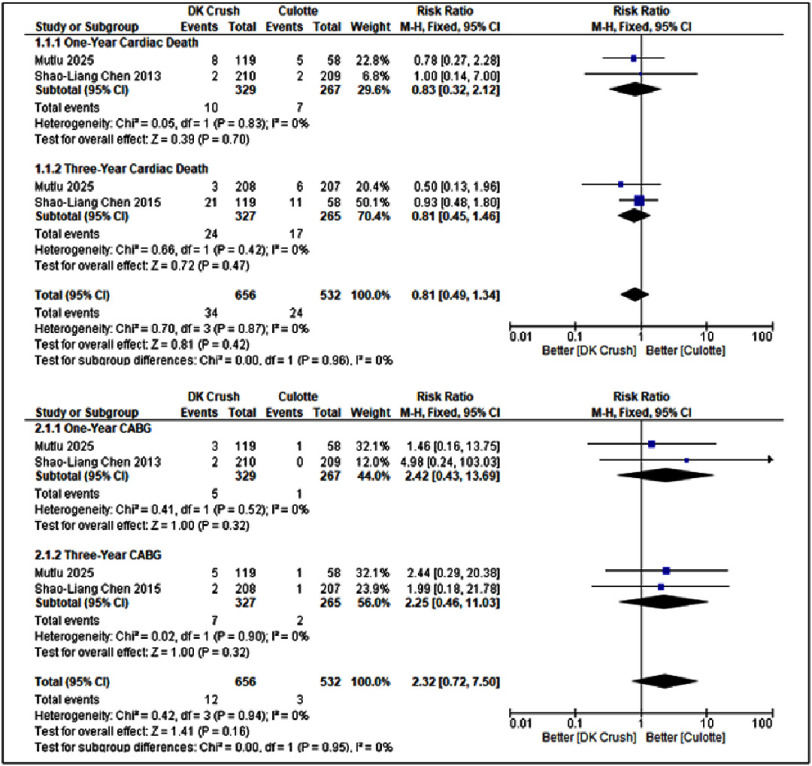
Forest plot of DK-Crush vs Culotte in Cardiac Death and CABG.

### Meta-analysis of left main coronary bifurcation stenting techniques: DK-Crush vs Culotte

#### Comparisons of DK-Crush vs Culotte in Cardiac Death and CABG

Figures 2 and 5 present the pooled and sensitivity analyses comparing the DK-Crush and Culotte techniques in patients with left main bifurcation lesions for the outcomes of cardiac death and coronary artery bypass grafting at one- and three-year follow-up. In the pooled analysis (Figure 2), there were no significant differences between DK-Crush and Culotte in terms of cardiac mortality or subsequent surgical revascularization. For one-year cardiac death (Panel A), the risk ratio (RR) was 0.83 (95% CI: 0.32–2.12, *p* = 0.70), indicating comparable short-term mortality between the two techniques. At the three-year follow-up (Panel B), the pooled RR was 0.81 (95% CI: 0.45–1.46, *p* = 0.47), again showing no statistically significant difference in long-term survival.

For CABG, the pooled one-year analysis (Panel C) demonstrated an RR of 2.42 (95% CI: 0.43–13.69, *p* = 0.32), and the three-year analysis (Panel D) yielded an RR of 2.25 (95% CI: 0.46–11.03, *p* = 0.32). Both results suggest that the requirement for subsequent surgical revascularization did not differ significantly between the DK-Crush and Culotte groups. Collectively, these findings indicate that both stenting strategies provide similar safety profiles regarding all-cause mortality and the need for CABG over both short- and long-term follow-up.

The sensitivity analysis (Figure 5) further supports the robustness of these findings. For cardiac death, exclusion of individual studies (Panels A and B) did not materially influence the pooled estimates. After omitting Mutlu 2025, the RR for one-year cardiac death was 1.00 [0.14–7.00], and after omitting Shao-Liang Chen 2013, the RR was 0.78 [0.27–2.28]. Similarly, at three years, the RR values were 0.93 [0.48–1.80] and 0.50 [0.13–1.96] following the respective exclusions. All *p*-values exceeded 0.3, confirming the stability of the overall results. A similar pattern was observed for CABG (Panels C and D). At one year, exclusion of Mutlu 2025 and Shao-Liang Chen 2013 yielded RR values of 4.98 [0.24–103.03] and 1.46 [0.16–13.75], respectively, while at three years, the corresponding RR values were 1.99 [0.18–21.78] and 2.44 [0.29–20.38]. None of these analyses reached statistical significance, and heterogeneity was negligible (I^2^ = 0%).

Overall, the sensitivity analyses confirmed the consistency and reliability of the pooled results. Both DK-Crush and Culotte demonstrated comparable performance in terms of cardiac death and the need for CABG at both one- and three-year follow-ups, without evidence of heterogeneity or instability across studies. These findings reinforce that the observed equivalence between the two stenting techniques is robust and not influenced by any single trial. Overall, Figure 2 demonstrates that DK-Crush did not confer a significant advantage over Culotte in reducing cardiac death or the need for CABG at either the one-year or three-year follow-ups. Both stenting strategies showed comparable safety profiles regarding mortality and surgical revascularization outcomes.

**Figure 3.**
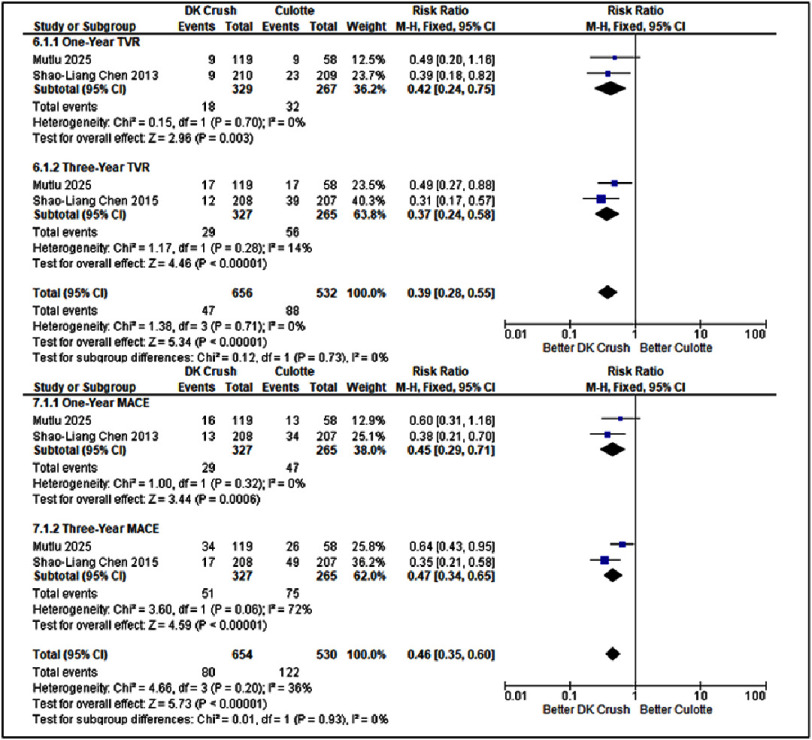
Forest plot of DK-Crush vs Culotte for TVR and MACE.

#### Comparisons of DK-Crush vs Culotte in TVR and MACE

Figures 3 and 6 present a comprehensive comparison between the DK-Crush and Culotte stenting techniques in patients with left main bifurcation lesions, focusing on target vessel revascularization (TVR) and major adverse cardiac events (MACE) at both one-year and three-year follow-ups. In the pooled analysis, DK-Crush consistently demonstrated better outcomes than Culotte across all time points. For TVR, the one-year pooled risk ratio (RR) was 0.42 (95% CI: 0.24–0.75, *p* = 0.003), and at three years, 0.37 (95% CI: 0.24–0.58, *p* < 0.0001). These findings indicate that DK-Crush significantly reduced the need for repeat revascularization both in the short and long term. The low level of heterogeneity (I^2^ = 0–14%) suggests that the observed benefit was consistent and not influenced by inter-study variation.

A similar advantage was observed for MACE outcomes. DK-Crush was associated with a markedly lower risk of MACE compared to Culotte, with a one-year pooled RR of 0.45 (95% CI: 0.29–0.71, *p* = 0.0006) and a three-year RR of 0.47 (95% CI: 0.34–0.65, *p* < 0.0001). This persistent benefit over time highlights the procedural advantages of DK-Crush in optimizing side-branch coverage and maintaining better flow dynamics at the bifurcation site. The absence of significant heterogeneity (I^2^ = 0–36%) further supports the stability and reproducibility of these findings across randomized controlled trials.

The sensitivity analysis presented in Figure 6 reinforced the reliability of the pooled results. For TVR, sequential exclusion of individual studies caused only minor changes in the overall effect. When Mutlu 2025 was excluded, the one-year pooled RR became 1.00 [0.40–2.46], *p* = 0.9917, and excluding Shao-Liang Chen 2013 yielded RR = 0.69 [0.33–1.44], *p* = 0.3186. The overall pooled estimate for one-year TVR remained RR = 0.80 [0.45–1.40], *p* = 0.4301, I^2^ = 0%, indicating no heterogeneity and stable results. For three-year TVR, exclusion of Mutlu 2025 produced RR = 0.31 [0.17–0.57], *p* = 0.0002, while removing Shao-Liang Chen 2015 yielded RR = 0.49 [0.27–0.88], *p* = 0.0178. The overall effect remained robust (RR = 0.37 [0.24–0.58], *p* < 0.0001, *I*^2^ = 11.4%) and statistically significant. A consistent trend was seen in the MACE sensitivity analyses. For the one-year outcome, exclusion of Mutlu 2025 resulted in RR = 0.38 [0.21–0.70], *p* = 0.0019, and omitting Shao-Liang Chen 2013 produced RR = 0.60 [0.31–1.16], *p* = 0.1298. The pooled estimate remained highly significant (RR = 0.45 [0.29–0.71], *p* = 0.0006, I^2^ = 0%). Similarly, for the three-year analysis, removing Mutlu 2025 resulted in RR = 0.35 [0.21–0.58], *p* < 0.0001, and excluding Shao-Liang Chen 2015 yielded RR = 0.64 [0.43–0.95], *p* = 0.0284. The overall pooled effect remained significant (RR = 0.47 [0.34–0.65], *p* < 0.0001, *I*^2^ = 70.2%). These findings confirm that the overall superiority of DK-Crush was not driven by any single study and that the results are consistent, reproducible, and statistically robust.

**Figure 4.**
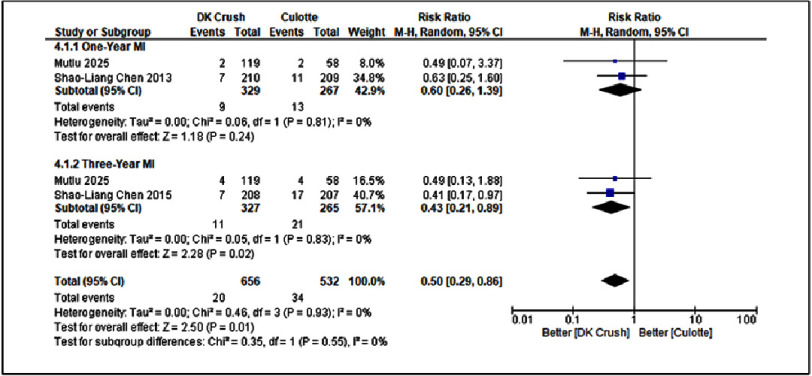
Forest plot of DK-Crush vs Culotte in MI.

**Figure 5.**
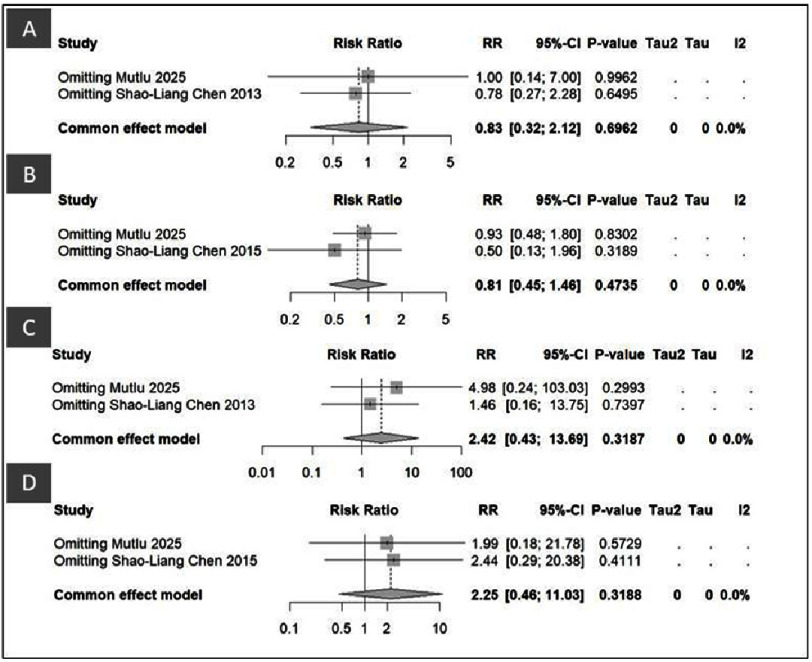
Sensitivity analysis of DK-Crush vs Culotte in (A) One-year Cardiac Death, (B) Three-year Cardiac Death, (C) One-year CABG, (D) Three-year CABG.

**Figure 6.**
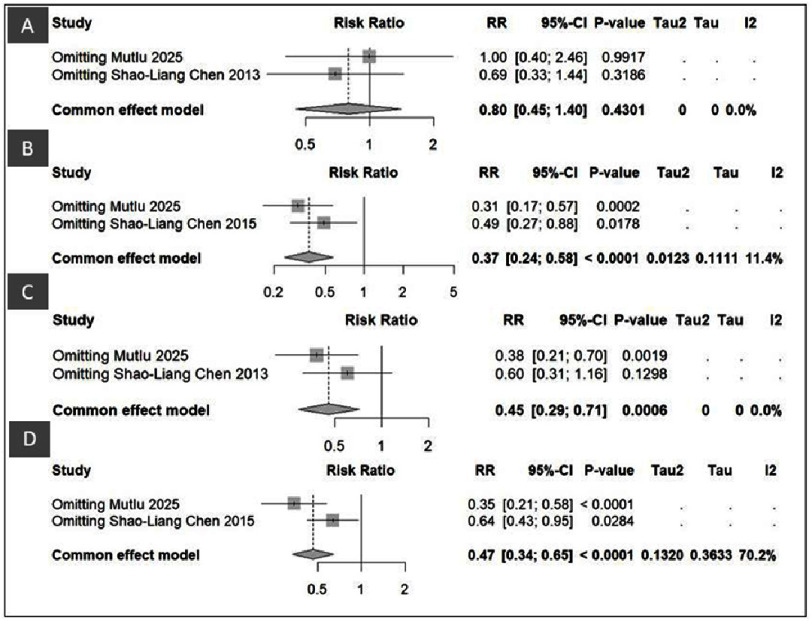
Sensitivity analysis of DK-Crush vs Culotte in (A) One-year TVR, (B) Three-year TVR, (C) One-year MACE, (D) Three-year MACE.

**Table 3:** Sensitivity analysis.

**Study Excluded**	**Outcome**	**Risk Ratio [95% CI]**	**p-value**
**Final outcome (all)**	Cardiac Death 1y	0.83 [0.32, 2.12]	0.6962
	Cardiac Death 3y	0.81 [0.45, 1.46]	0.4735
	CABG 1y	2.42 [0.43, 13.69]	0.3187
	CABG 3y	2.25 [0.46, 11.03]	0.3188
	MI 1y	0.60 [0.26, 1.39]	0.2400
	MI 3y	0.43 [0.21, 0.89]	0.0200
	MACE 1y	0.45 [0.29, 0.71]	0.0006
	MACE 3y	0.47 [0.34, 0.65]	0.0001
	TVR 1y	0.80 [0.45, 1.40]	0.4301
	TVR 3y	0.37 [0.24, 0.58]	0.0001
**Omit Mutlu 2025**	Cardiac Death 1y	1.00 [0.14, 7.00]	0.9962
	Cardiac Death 3y	0.93 [0.48, 1.80]	0.8302
	CABG 1y	4.98 [0.24, 103.03]	0.2993
	CABG 3y	1.99 [0.18, 21.78]	0.5729
	MI 1y	0.49 [0.07, 3.37]	0.8110
	MI 3y	0.49 [0.13, 1.88]	0.3189
	MACE 1y	0.38 [0.21, 0.70]	0.0019
	MACE 3y	0.35 [0.21, 0.58]	0.0001
	TVR 1y	1.00 [0.40, 2.46]	0.9917
	TVR 3y	0.31 [0.17, 0.57]	0.0123
**Omit Shao-Liang Chen**	Cardiac Death 1y	0.78 [0.27, 2.28]	0.6495
	Cardiac Death 3y	0.50 [0.13, 1.96]	0.3189
	CABG 1y	1.46 [0.16, 13.75]	0.7397
	CABG 3y	2.44 [0.29, 20.38]	0.4111
	MI 1y	0.63 [0.25, 1.60]	0.4300
	MI 3y	0.41 [0.17, 0.97]	0.0411
	MACE 1y	0.60 [0.31, 1.16]	0.1298
	MACE 3y	0.64 [0.43, 0.95]	0.0284
	TVR 1y	0.69 [0.33, 1.44]	0.3188
	TVR 3y	0.49 [0.27, 0.88]	0.0178

#### Comparisons of DK-Crush vs Culotte in MI

Figure 4 presents the pooled analysis comparing the DK-Crush and Culotte techniques in terms of myocardial infarction (MI) at one- and three-year follow-ups. At the one-year mark, DK-Crush demonstrated a non-significant trend toward reducing MI risk, with a risk ratio (RR) of 0.60 [95% CI: 0.26–1.39], *p* = 0.24. However, by the three-year follow-up, this difference reached statistical significance, showing a RR of 0.43 [95% CI: 0.21–0.89], *p* = 0.02. These results indicate that while the short-term effect was not significant, DK-Crush was associated with a substantially lower risk of MI in the long term. The overall pooled analysis yielded a RR of 0.50 [95% CI: 0.29–0.86], with no heterogeneity (I^2^ = 0%), suggesting consistency across the included studies.

**Figure 7.**
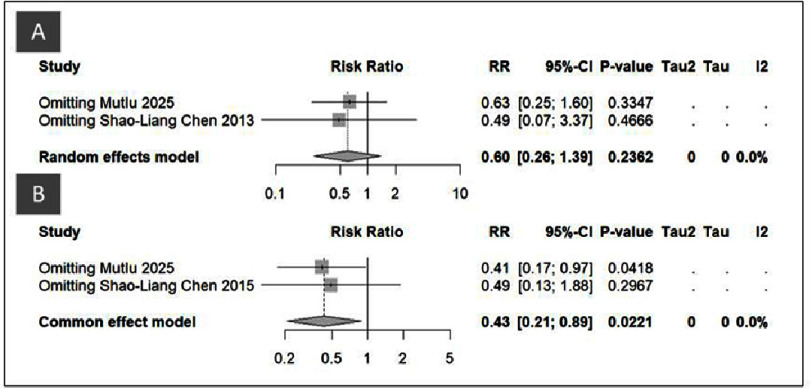
Sensitivity analysis of DK-Crush vs Culotte in (A) One-year MI, (B) Three-year MI.

The corresponding sensitivity analysis (Figure 7, Table 3) confirmed the robustness of these findings. For one-year MI, exclusion of Mutlu 2025 resulted in RR = 0.63 [0.25–1.60], *p* = 0.33, and omitting Shao-Liang Chen 2013 produced RR = 0.49 [0.07–3.37], *p* = 0.47. The pooled effect remained consistent (RR = 0.60 [0.26–1.39], *p* = 0.24, I^2^ = 0%), demonstrating stable results without evidence of heterogeneity. For three-year MI, excluding Mutlu 2025 yielded RR = 0.41 [0.17–0.97], *p* = 0.04, while removal of Shao-Liang Chen 2015 gave RR = 0.49 [0.13–1.88], *p* = 0.30. The overall pooled estimate remained statistically significant (RR = 0.43 [0.21–0.89], *p* = 0.02, I^2^ = 0%). These findings confirm that the observed advantage of DK-Crush in reducing long-term MI risk was not driven by any single study, reinforcing the reliability and internal validity of the meta-analytic results.

## Discussion

This meta-analysis included three randomized controlled trials (RCTs) encompassing 1,188 patients with left main bifurcation lesions (LMBLs), of whom 656 underwent DK-Crush and 532 underwent Culotte stenting. The analysis compared both short- and long-term outcomes at one- and three-year follow-ups.

The pooled results showed that DK-Crush significantly reduced the risks of target vessel revascularization, major adverse cardiac events, and myocardial infarction, while rates of cardiac death and coronary artery bypass grafting were comparable between the two techniques. Specifically, DK-Crush lowered TVR at one year with a pooled risk ratio (RR) of 0.42 [95% CI: 0.24–0.75; *p* = 0.003] and at three years with RR 0.37 [95% CI: 0.24–0.58; *p* < 0.0001]. Similarly, MACE was significantly reduced in the DK-Crush group, with RR 0.45 [95% CI: 0.29–0.71; *p* = 0.0006] at one year and RR 0.47 [95% CI: 0.34–0.65; *p* < 0.0001] at three years.

The heterogeneity for these outcomes was low (I^2^ = 0–36%), indicating high consistency across studies^3,13,14^. These findings are consistent with other studies, such as those by Chen et al. (2019), who reported similar outcomes for TVR and TLR between DK-Crush and Culotte but a trend favoring DK-Crush for lower stent thrombosis and myocardial infarction rates^3,15,16^.

Similarly, a study by Mutlu et al 2025 revealed that, compared with Culotte, DK-Crush had a significantly lower rate of major adverse cardiac events (MACE) during long-term follow-up. Although some studies, such as the one by Yıldız et al. (2024), reported a trend favoring DK-Crush, the results were not statistically significant for all outcomes at three years^13,17^. Furthermore, other investigations, such as those of Chen et al 2020 and Mutlu et al 2025, suggested that while there are slight differences in outcomes, particularly in terms of stent thrombosis and MI, neither technique exhibited significant superiority in long-term outcomes^3,6,18^. These results highlight the need for further research with larger cohorts and extended follow-up periods to conclusively determine the optimal strategy for treating complex bifurcation lesions.

For myocardial infarction, DK-Crush was associated with a trend toward lower events at one year (RR 0.60 [95% CI 0.26–1.39]; *p* = 0.24) and achieved a statistically significant benefit at three years (RR 0.43 [95% CI: 0.21–0.89]; *p* = 0.02). Overall, the pooled estimate confirmed a 50% reduction in MI risk (RR 0.50 [95% CI: 0.29–0.86]), with no heterogeneity (I^2^ = 0%), suggesting that this benefit was consistent and reproducible. In contrast, there were no significant differences in cardiac death or CABG between the two strategies. For cardiac death, the pooled one-year RR was 0.83 [95% CI: 0.32–2.12]; *p* = 0.70, and the three-year RR was 0.81 [95% CI: 0.45–1.46]; *p* = 0.47. Similarly, for CABG, the pooled RRs were 2.42 [95% CI: 0.43–13.69]; *p* = 0.32 at one year and 2.25 [95% CI: 0.46–11.03]; *p* = 0.32 at three years. These findings demonstrate that while DK-Crush effectively reduces ischemic and repeat revascularization events, both techniques are equally safe in terms of long-term survival and the need for surgical revascularization^1^. A key advantage of the DK Crush technique lies in the immediate first kissing balloon inflation after crushing, which enhances stent expansion. One key advantage of the DK crush technique is the immediate first kissing balloon inflation after crushing, followed by high-pressure noncompliant balloon inflation, final kissing, and proximal optimization, which together enhance stent expansion and apposition^19^.

Sensitivity analyses further validated the robustness of these results. Sequential omission of individual studies produced only minor variations in pooled risk ratios and did not alter statistical significance. For example, in the leave-one-out analysis, the overall one-year TVR remained RR 0.80 [95% CI [0.45–1.40]]; *p* = 0.43, and the three-year TVR remained RR 0.37 [95% CI: 0.24–0.58]; *p* < 0.0001. Similarly, for MACE, exclusion of any single trial yielded consistent results, with the one-year RR remaining 0.45 [95% CI: 0.29–0.71]; *p* = 0.0006 and the three-year RR 0.47 [95% CI: 0.34–0.65]; *p* < 0.0001. For MI, the stability of results was also confirmed (three-year RR = 0.43 [95% CI: 0.21–0.89]; *p* = 0.02; I^2^ = 0%). These consistent findings across all sensitivity models reinforce the reliability and internal validity of the meta-analytic conclusions.

Notably, these results differ somewhat from earlier trials—such as DKCRUSH-III and DKCRUSH-V—which reported clear superiority of DK-Crush in reducing long-term MACE and stent thrombosis^1,12^. Several explanations may account for this discrepancy. The included RCTs in our analysis enrolled patients with varying bifurcation complexity and lesion morphology. For example, some studies included a mix of true and non-true bifurcation lesions (Medina 1,1,1 vs. 0,1,1), which could attenuate the comparative advantage of DK-Crush. Furthermore, operator expertise plays a critical role: DK-Crush requires meticulous execution of multiple procedural steps—two kissing balloon inflations, proximal optimization, and precise wire recrossing—which demand substantial experience^14,20^. Differences in operator proficiency and center experience may have influenced outcomes, particularly in less specialized centers. Variations in follow-up duration, sample size, and endpoint definitions across trials could also explain why the mortality and CABG outcomes did not achieve statistical significance despite numerically favorable trends.

From a clinical perspective, the comparable rates of cardiac death and CABG between the two groups suggest that both techniques offer similar safety profiles when performed properly. However, DK-Crush demonstrated superior efficacy in minimizing repeat revascularization and ischemic complications, likely owing to its ability to ensure more complete side-branch coverage and optimal stent apposition^21^. For patients with complex, true bifurcation lesions and large side branches, DK-Crush may therefore be the preferred approach. Conversely, in less complex bifurcations or settings with limited operator experience, the Culotte technique remains a reasonable and technically simpler alternative that provides acceptable outcomes with shorter procedural and fluoroscopy times^22,23^. Thus, the choice between the two strategies should be guided by lesion anatomy, operator skill, and institutional experience rather than a universal hierarchy of procedural superiority.

This study offers several strengths, including the exclusive inclusion of RCTs, consistent follow-up intervals, and comprehensive sensitivity analyses confirming the reproducibility of results. Nonetheless, limitations should be acknowledged. The small number of studies (*n* = 3) and modest sample size may limit the statistical power to detect rare outcomes such as cardiac death. Heterogeneity in patient selection, procedural execution, and post-PCI management introduces potential bias. Additionally, angiographic and intravascular imaging data were insufficient for assessing stent expansion or apposition differences. The certainty of evidence, assessed using the GRADE framework, ranged from low to moderate, primarily due to small sample sizes, some risk of bias, and imprecision. Future large-scale multicenter RCTs with standardized procedural protocols and imaging follow-up are warranted to validate these findings and refine recommendations for complex left main bifurcation PCI.

In clinical practice, both DK-Crush and Culotte are viable options for two-stent treatment of left main bifurcation lesions. DK-Crush should be favored for complex, true bifurcations requiring durable side-branch patency and minimized ischemic risk, provided it is performed by experienced operators. Culotte remains a practical choice in less complex anatomies or when procedural simplicity and time efficiency are paramount. Ultimately, individualized strategy selection, guided by bifurcation anatomy, operator expertise, and institutional familiarity, offers the best pathway to optimizing long-term patient outcomes.

## Conclusions

In summary, this meta-analysis shows that the DK-Crush stenting technique delivers better long-term outcomes than the Culotte technique in patients with left main bifurcation lesions. DK-Crush was associated with fewer cases of target vessel revascularization, major adverse cardiac events, and myocardial infarction at both one and three years of follow-up, while rates of cardiac death and coronary artery bypass grafting were similar between the two approaches. Although DK-Crush is technically more complex and requires longer procedure and fluoroscopy times, it consistently achieved high procedural success across all studies. These findings suggest that DK-Crush offers a more durable and effective treatment option for complex left main bifurcation disease, providing sustained clinical benefits without sacrificing procedural safety.

## Conflict of interest

The authors declare that there are no conflicts of interest.

## Funding

The authors received no financial support for their research, authorship, or publication of this article.

## Ethical Approval

Not Applicable.

## Informed Consent

Not Applicable.

## Abbreviations

CABG, coronary artery bypass grafting; CD, cardiac death; CI, confidence interval; DK, double kissing; MACE, major adverse cardiac events; MI, myocardial infarction; PCI, percutaneous coronary intervention; PRISMA, Preferred Reporting Items for Systematic Reviews and Meta-Analyses; PROSPERO, International Prospective Register of Systematic Reviews; RCT, randomized controlled trial; RR, risk ratio; TVR, target vessel revascularization.
